# S16-1 Get Finland Moving programme

**DOI:** 10.1093/eurpub/ckae114.270

**Published:** 2024-09-26

**Authors:** Päivi Aalto-Nevalainen

**Affiliations:** Division for Sport, Ministry of Education and Culture, Finland

## Abstract

**Purpose:**

With the introduction of the new governmental programme, the Finnish Government targets to increase physical activity across all age groups. To accomplish this goal, a comprehensive cross-administrative initiative called the 'Get Finland Moving' programme has been initiated, aiming to promote physically active lifestyles. The program is grounded in phenomenon-based thinking and leveraging a thorough evaluation of the branches within each of the 12 Ministries. This evaluation assessed each Ministrýs relationships, roles, and opportunities to promote physical activity within society.

**Project or policy description:**

Over the past years, Finland has developed a comprehensive, well-managed package dedicated to promoting physical activity across all age groups. The 'Get Finland Moving' programme is poised to further fortify and expand the cross-administrative initiatives aimed at increasing physical activity. The government executes the action plan through various measures, such as reinforcing physically active practices in early childhood education, schools, and educational institutes. Additionally, integrating physical activity into the development of work ability and workplaces is a key focus. Steps will be taken to enhance physical activity promotion skills and competence in different educational sectors, including healthcare, social welfare, and education sciences. Efforts will also be directed towards improving lifestyle counselling and physical activity guidance in municipalities and well-being service counties.

Furthermore, initiatives include adding a ball to the maternity pack and increasing the provision of advice on physical activity for families at maternity and child health clinics. The promotion of physically active lifestyles will be incorporated into the Act on Primary and Lower Secondary Education, as well as in the reform of land use legislation. Additionally, measures involve exploring and evaluating options for promoting physical activity through taxation and examining the feasibility of guaranteeing a subjective right to outdoor recreation for older people.

**Conclusion:**

One distinctive aspect of the programme is its management and monitoring at the government level, with measures intricately aligned with the diverse administrative branches of various ministries. Another noteworthy feature involves a meticulous analysis of how different administrative branches are connected to the promotion of physical activity.

